# Contact with adult hen affects development of caecal microbiota in newly hatched chicks

**DOI:** 10.1371/journal.pone.0212446

**Published:** 2019-03-06

**Authors:** Tereza Kubasova, Miloslava Kollarcikova, Magdalena Crhanova, Daniela Karasova, Darina Cejkova, Alena Sebkova, Jitka Matiasovicova, Marcela Faldynova, Alexandra Pokorna, Alois Cizek, Ivan Rychlik

**Affiliations:** 1 Veterinary Research Institute, Brno, Czech Republic; 2 Department of Infectious Diseases and Microbiology, Faculty of Veterinary Medicine, University of Veterinary and Pharmaceutical Sciences Brno, Brno, Czech Republic; 3 Central European Institute of Technology (CEITEC), University of Veterinary and Pharmaceutical Sciences Brno, Brno, Czech Republic; University of Illinois, UNITED STATES

## Abstract

Chickens in commercial production are hatched in a clean hatchery environment in the absence of any contact with adult hens. However, *Gallus gallus* evolved to be hatched in a nest in contact with an adult hen which may act as a donor of gut microbiota. In this study, we therefore addressed the issue of microbiota development in newly hatched chickens with or without contact with an adult hen. We found that a mere 24-hour-long contact between a hen and newly hatched chickens was long enough for transfer of hen gut microbiota to chickens. Hens were efficient donors of *Bacteroidetes* and *Actinobacteria*. However, except for genus *Faecalibacterium* and bacterial species belonging to class *Negativicutes*, hens did not act as an important source of Gram-positive *Firmicutes*. Though common to the chicken intestinal tract, *Lactobacilli* and isolates from families *Erysipelotrichaceae*, *Lachnospiraceae* and *Ruminococcaceae* therefore originated from environmental sources instead of from the hens. These observation may have considerable consequences for the evidence-based design of the new generation of probiotics for poultry.

## Introduction

The microbial community in the distal parts of the intestinal tract of adult warm-blooded animals consists of up to 10^10^ bacterial cells per gram of digesta. The environment is anaerobic with a stable temperature and continuous nutrient supply. Although it is estimated that approx. 1,000 different bacterial species comprise the microbiota of the intestinal tract, these belong to two main phyla only; Gram-positive *Firmicutes* and Gram-negative *Bacteroidetes*. Representatives of these two phyla commonly form around 95% of the total gut microbiota in healthy adults. The remaining 5% of microbiota is formed by representatives of *Proteobacteria* and *Actinobacteria*, followed by minority microbiota members of phyla *Verrucomicrobia*, *Synergistetes*, *Deferribacteres*, *Fusobacteria*, *Spirochaetes* and some others [1–3].

Chickens evolved for millions of years to be hatched in a nest in contact with an adult hen. On the other hand, current commercial production of chickens is based on hatching chicks in a clean hatchery environment in the absence of adult hens. Colonisation of commercially hatched chickens is therefore exclusively dependent on environmental sources during which the caecum of chickens is first colonised by *Enterobacteriaceae* (phylum *Proteobacteria*), which are replaced by *Lachnospiraceae* and *Ruminococcaceae* (phylum *Firmicutes*) during the second week of life. At around one month of age, *Firmicutes* become complemented by bacterial isolates belonging to phylum *Bacteroidetes* [2]. This gradual microbiota development during the first weeks of life leaves chicks highly susceptible to different infections, *e*.*g*. with *Salmonella* [4, 5] although it is well established that inoculation of chicks with microbiota of adult hens can increase their resistance to *Salmonella* [4, 6, 7]. Despite this, studies focused on the microbiota transfer between hens and chicks are absent. It is not known whether all microbiota members or only a certain subset of microbiota is effectively transferred from hens to chicks. It is also not known, how rapid the transfer of microbiota between the hen and chicks is, *i*.*e*. whether the contact between the hen and chicks must last for a day, a week, a month or even longer. Considering both the biological significance and economic consequences, it is rather surprising that microbiota transfer between a hen and chicks has not been addressed in necessary detail so far especially when recent developments in next generation sequencing allow for the topic to be addressed directly. Obtained knowledge can be then used to identify bacterial genera which are efficiently transferred from hens to chicks followed by their isolation in pure culture. Administration of pure cultures of such isolates or their mixtures should then mimic the natural transfer from a hen to chicks and improve gut health of the chicks from the very first days of life. However, this can be achieved only using evidence-based approach reflecting principles of natural microbiota transfer between a hen and a chick.

In this study, we therefore addressed the rate and efficiency of microbiota transfer between hens and chicks. Microbiota transfer was modelled by cohabiting newly hatched chicks in a single space with an adult hen. We found that hens acted as the donors of *Bacteroidetes* and *Actinobacteria*, but rather unexpectedly, except for genus *Faecalibacterium* and bacterial species belonging to class *Negativicutes*, hens did not act as an important source of Gram-positive *Firmicutes*.

## Materials and methods

### Ethical statement

The handling of animals in the study was performed in accordance with current Czech legislation (Animal Protection and Welfare Act No. 246/1992 Coll. of the Government of the Czech Republic). The specific experiments were approved by the Ethics Committee of the Veterinary Research Institute followed by the Committee for Animal Welfare of the Ministry of Agriculture of the Czech Republic (permit number MZe1922). Since chickens do not die after inoculation with gut microbiota and/or *Salmonella*, and even do not experience any discomfort, length of the experiments was defined only by experimental needs specified below. Chickens were routinely daily monitored for unexpected behaviour what confirmed absence of any abnormal behaviour or even fatalities.

### Experimental chickens and *Salmonella* used for challenge

In all experiments, newly hatched male ISA Brown chicks were obtained from a local commercial hatchery on the day of hatching. Contact Lohmann Brown hens acting as a natural source of gut microbiota were obtained from a local commercial egg laying hen farm. Donor hens originated from enriched cages and had no access to outdoor environment at any time during rearing period. Chicks were reared in perforated plastic boxes of 2 m^2^ with free access to water and standard starter feed, *i*.*e*. not sterilised. No specific feed additives or therapeutics, *e*.*g*. coccidiostatics, were used in these experiments. Temperature was set to 30°C during the first week of life and to 28°C in the second week of life. Light regime was set up to 24 hours light in the first week of life and 22 hours of light during the second week of life. When chicks were challenged with *Salmonella*, the infection was performed orally with 1 x 10^7^ CFU *Salmonella* Enteritidis 147 spontaneously resistant to nalidixic acid in 0.1 ml inoculum [8]. All chicks were sacrificed under chloroform anesthesia by cervical dislocation and during necropsy, 0.5 g liver and cecum were collected to enumerate *S*. Enteritidis. Aliquots of caecal contents were collected and frozen at -20°C within 10 min after collection for microbiota characterisation.

### Microbiota transfer by contact

**Fig 1:**
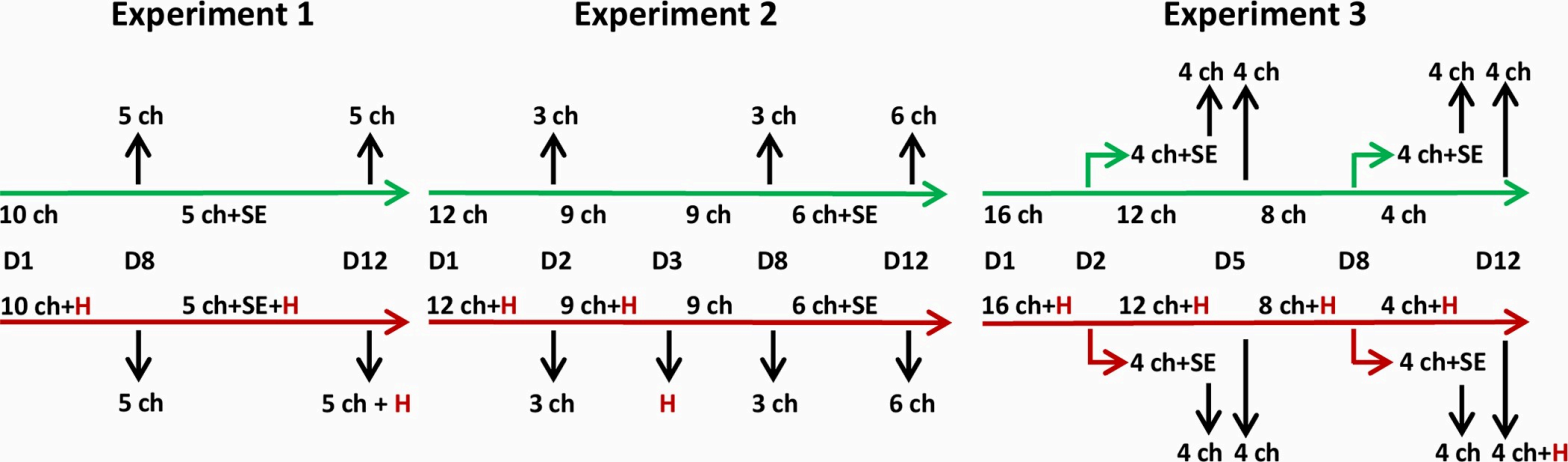
Design of the experiments with contact hens. Number of chicks in control and experimental groups, date of intervention or sample collection, transfer of chicks into a new clean room in experiment 3 and numbers of chicks remaining in the experiments are shown for each of the experiments with contact hens. Ch–chicks, H–hen, SE–infection with *S*. Enteritidis, D–age of chicks in days.

In the first experiment, 20 newly hatched chicks were divided into 2 groups. Ten chicks in the experimental group were reared in a single cage together with a 45-week-old hen. Chicks in the control group were kept under the same conditions but without any contact with an adult hen. Seven days later, five chicks from both groups were sacrificed to check for caecal microbiota composition and the remaining 5 chicks in each group were orally challenged with *S*. Enteritidis. All infected chicks and the contact donor hen were sacrificed 4 days later (Fig 1).

In the second experiment, 24 newly hatched chicks were divided into 2 groups. Twelve chicks in the experimental group were reared in a single cage with a 34-week-old hen. Chicks in the control group were kept under the same conditions but without any contact with an adult hen. Unlike the previous experiment, 3 chicks from each group were sacrificed on day 2 of life, *i*.*e*. after 24-hour-long contact with an adult hen. The donor hen was removed from the contact chicks an additional 24 hours later, *i*.*e*. when the chicks were 3 days old. The rest of the experiment followed the format of the first experiment, *i*.*e*. three chicks from each group were sacrificed on day 7 of life and the remaining 6 chicks in each group were orally challenged with *S*. Enteritidis. All infected chicks were sacrificed 4 days later (Fig 1).

In the last experiment targeted to natural microbiota transfer, 32 newly hatched chicks were divided into 2 groups. Sixteen chicks in the experimental group were reared in a single cage with a 34-week-old hen. On day 2 of life, *i*.*e*. only after 24 hour contact with an adult hen, four chicks from the contact and control groups were moved to two separate rooms where they were orally infected with *S*. Enteritidis. This was performed primarily to prevent *Salmonella* infection in the rest of the chicks but served also to test whether a mere 24-hour-long contact followed by additional 3 days of life would enable microbiota transfer and development between the hen and chicks. Three days later, *i*.*e*. on day 5 of life, the infected chicks were sacrificed together with an additional 4 non-infected chicks from the control group and 4 non-infected chicks from the contact group. Next, four chicks from both groups were moved to two separate rooms where they were orally challenged with *S*. Enteritidis. Four days later the experiment was terminated and infected and non-infected chicks together with the donor hen were sacrificed (Fig 1).

### Colonisation of newly hatched chicks with bacterial cultures of moderate complexity

The colonisation of newly hatched chicks was tested also with bacterial populations of moderate complexity. This was achieved by chick inoculation with Aviguard, commercially available competitive exclusion product containing the normal gut microbiota from specific pathogen-free chickens (Lallemand, France) or by bacterial washes from WCHA (Wilkins-Chalgren Agar) and YCFA (Yeast extract, Casitone and Fatty Acid agar) agars obtained by anaerobic culture of serially diluted caecal samples from an adult hen. For bacterial composition of these inocula as well as of the batch of the Aviguard used in this study, see S1 Table.

In the experiment with Aviguard, 24 newly hatched chicks were divided into 2 groups. Chicks in the experimental group were orally inoculated on day 1 of life with 100 μl of Aviguard solution prepared according to the recommendations of the manufacturer. Chicks in the control group were kept under the same conditions but without any treatment. On day 7 of life, 6 chicks in both groups were sacrificed to check for caecal microbiota composition. The remaining 6 chicks in each group were infected with *S*. Enteritidis and 4 days later, the experiment was terminated.

### Inoculation with bacterial mass collected from WCHA and YCFA agars

To obtain bacterial mass growing on WCHA and YCFA agars, the caecal content of a 45-week-old hen was resuspended in pre-reduced anaerobically sterilised dilution blank (PRAS—0.1 g magnesium sulfate heptahydrate, 0.2 g monobasic potassium phosphate, 0.2 g potassium chloride, 1.15 g dibasic sodium phosphate, 3.0 g sodium chloride, 1.0 g sodium thioglycolate, 0.5 g L-cysteine, 1000 ml distilled water, pH 7.5 at 25°C), tenfold serially diluted and plated on WCHA or YCFA agars. The agar plates were incubated in an anaerobic chamber (10% CO_2_, 5% H_2_ and 85% N_2_ atmosphere; Concept 400, Baker Ruskinn, USA) at 37°C for 3 days. The bacterial mass was washed with 2 x 2 ml of PRAS solution from agar plates on which approx. 500 colonies grew. The obtained suspension was split into two aliquots. The first one was frozen at -20°C for characterisation of microbial composition by sequencing over V3/V4 variable regions of 16S rRNA genes and the second one was immediately used for oral inoculation of newly hatched chicks. Altogether, 30 newly hatched chicks were divided into 3 groups. Ten chicks were orally inoculated with 0.1 ml of suspension collected from WCHA agar, another group of 10 chicks was inoculated with the suspension collected from YCFA agar and the last 10 chicks served as a non-inoculated control. The control chicks were the same as those used in the first experiment with the contact hen as these experiments were carried out at the same time to reduce the number of chicks used. Seven days later, five chicks from each group were sacrificed to check for caecal microbiota composition and the remaining 5 chicks in each group were orally challenged with *S*. Enteritidis. All chicks were sacrificed 4 days later and *S*. Enteritidis counts were determined as described previously [9].

### Colonisation of newly hatched chicks with pure cultures of selected gut anaerobes

Finally, we inoculated newly hatched chicks with pure cultures of selected gut anaerobes [10]. We deliberately selected a phylogenetically broad spectrum of bacterial species. Groups of three newly hatched chicks were inoculated with 10^7^ CFU in 0.1 ml of inoculum containing *Parabacteroides johnsonii* An42 (phylum *Bacteroidetes*, GenBank access. n. NFIJ00000000) or *Bacteroides clarus* An43 (*Bacteroidetes*, NFII00000000) as representatives of Gram-negative bacteria, or with *Megamonas hypermegale* An288, (*Selenomonadales*/*Firmicutes*, NFIW00000000), *Lactobacillus reuteri* An71 (*Lactobacillaceae*/*Firmicutes*, NFHN00000000), *Butyricicoccus pullicaecorum* An179 (*Ruminococcaceae*/*Firmicutes*, NFKL00000000) or *Blautia producta* An81 (*Lachnospiraceae*/*Firmicutes*, NFKQ00000000) as representatives of Gram-positive bacteria. On day 7, all 3 chicks from each group were sacrificed to check for caecal microbiota composition and for the presence of the strains used for inoculation.

### Sequencing of V3/V4 region of 16S rRNA genes

Caecal content samples were homogenised in a MagNALyzer (Roche). Following homogenisation, the DNA was extracted using a QIAamp DNA Stool Mini Kit according to the manufacturer’s instructions (Qiagen). The DNA concentration was determined spectrophotometrically and DNA samples diluted to 5 ng/ml were used as a template in PCR with forward primer 5´- *TCGTCGGCAGCGTCAGATGTGTATAAGAGACAG*-MID-GT-CCTACGGGNGGCWGCAG-3´ and reverse primer 5´-*GTCTCGTGGGCTCGGAGATGTGTATAAGAGACAG*-MID-GT GACTACHVGGGTATCTAATCC-3´.

The sequences in italics served for index and adapter ligation whereas the underlined sequences allowed for the amplification over the V3/V4 region of 16S rRNA genes as recommended by Illumina. MIDs represent different sequences of 5, 6, 7, or 9 base pairs in length which were used to identify individual samples within the sequencing groups. PCR amplification was performed using a HotStarTaq Plus MasterMix kit. The resulting PCR products were purified using AMPure beads. In the next step, the concentration of PCR products was determined spectrophotometrically, the DNA was diluted to 100 ng/μl and groups of 14 PCR products with different MID sequences were indexed with a Nextera XT Index Kit following the manufacturer’s instructions (Illumina). Prior to sequencing, the concentration of differently indexed samples was determined using a KAPA Library Quantification Complete kit (Kapa Biosystems). All indexed samples were diluted to 4 ng/μl and 20 pM phiX DNA was added to final concentration of 5% (v/v). Sequencing was performed using MiSeq Reagent Kit v3 (600 cycle) and MiSeq apparatus according to the manufacturer’s instructions (Illumina).

Quality trimming of the raw reads was performed using TrimmomaticPE v0.32 with sliding window 4 bp and quality read score equal or higher than 15 [11]. Minimal read length must have been at least 150 bp. The fastq files generated after quality trimming were uploaded into QIIME software [12]. Forward and reverse sequences were joined and in the next step, chimeric sequences were predicted and excluded by the slayer algorithm. The resulting sequences were then classified by RDP Seqmatch with an OTU (operational taxonomic units) discrimination level set to 97%. Principal coordinate analysis (PCoA) implemented in QIIME was used for data visualisation. The raw sequence reads were deposited in the NCBI Short Read Archive under accession number PRJNA489774 (SRP161500).

### Statistics and reproducibility

The significance of the differences between the microbiota composition in the control and contact chickens was determined by Mann-Whitney U test using percentage representation of individual genera for ranking in the non-parametric Mann-Whitney U test. Since the results of this study could have been affected by differences in the microbiota composition of contact hens used on 3 different and independent occasions, the statistical analysis was performed at genus and not at OTU level over the data collected in all 3 independent experiments. In addition to statistical significance, differentially abundant genera must have been present in at least 0.5% average abundance in microbiota of either control or contact chicks, and the difference in abundance in control and contact chicks must have been 5 fold or higher. *Salmonella* counts in contact and appropriate control chicks were compared by t-test. In all cases, comparisons with p values lower than 0.05 were considered significant.

## Results

### Sequencing parameters

In total 6,333,147 sequence reads were obtained for 125 samples analysed in this study. The average coverage per sample was 50,665 reads with a minimal and maximal sample read coverage ranging from 15,388 to 148,843, respectively.

### Microbiota transfer by contact

**Fig 2:**
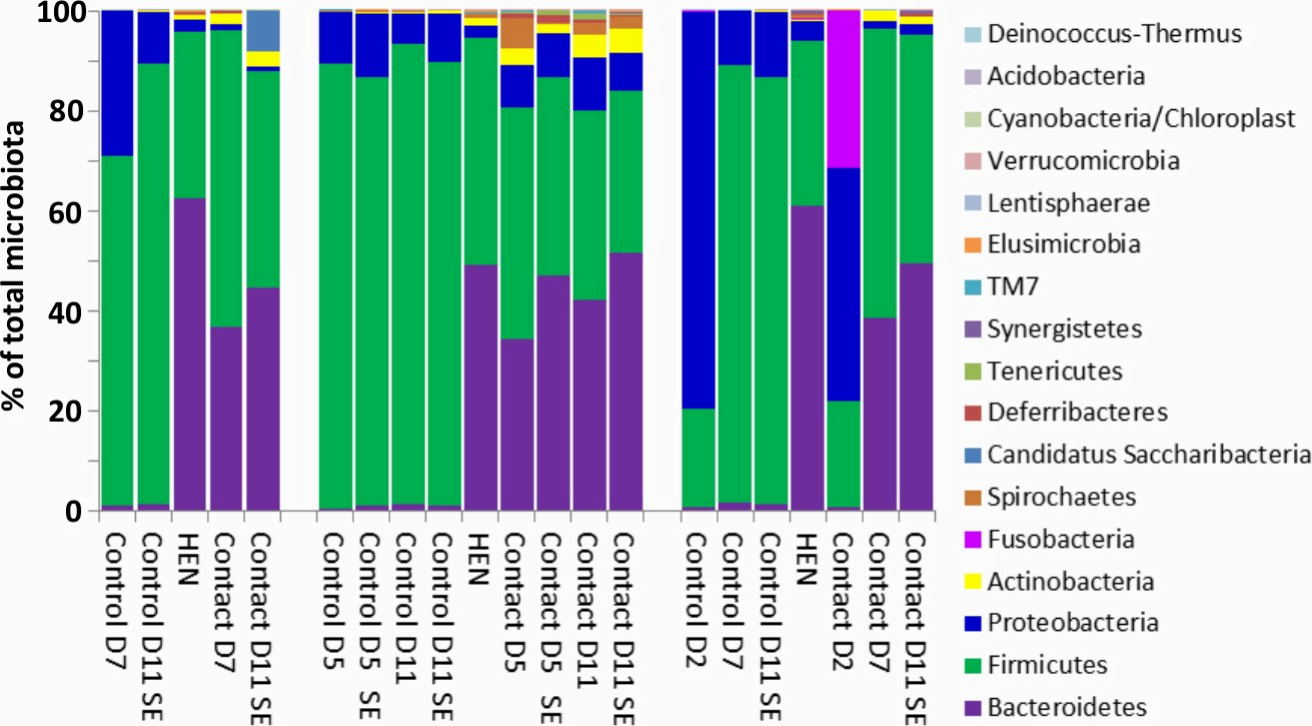
Composition of caecal microbiota of individual chicks and donor hens at phylum level. Age when sacrificed in days is shown for control or contact chicks. SE–*S*. Enteritidis infected chickens. *S*. Enteritidis infection is of low effect on microbiota composition in chickens not detectable at phylum level [13, 14]. Average abundance of each phylum recorded in three independent experiments is shown.

Three independent experiments with chicks raised in the presence or absence of a contact hen showed extensive differences in the composition of caecal microbiota between the control and contact chicks. Microbiota of control chicks raised in the absence of a hen was dominated by Gram-positive representatives of phylum *Firmicutes*. On the other hand, approx. 40% of the caecal microbiota of contact chicks was formed by Gram-negative representatives of phylum *Bacteroidetes*. Adult hens also acted as donors of *Actinobacteria* (Fig 2 and S1 Table). The 24-hour-long contact with an adult hen was long enough for the inoculation of the chicks as the chicks which were transferred to another room after 24-hour long contact with a hen developed complex microbiota on day 5 of life in the Experiment 2 (Fig 2). However, a period longer than 24 hours was required for the microbiota to develop to the composition observed in 5-day-old or older chicks since the microbiota in 2-day-old contact chicks in Experiment 3 did not yet contain a high level of *Bacteroidetes* and *Actinobacteria* (Fig 2).

**Fig 3:**
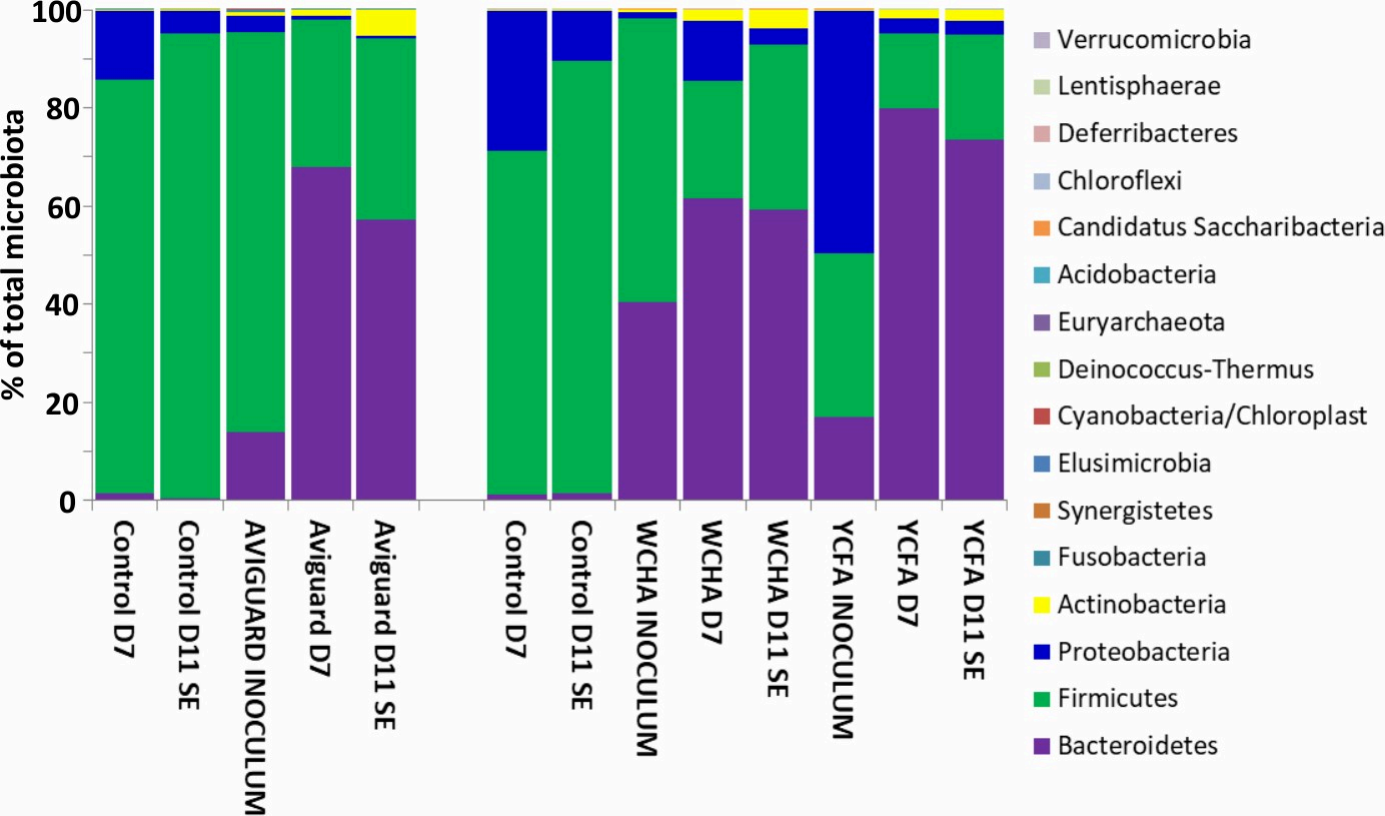
Composition of caecal microbiota of individual chicks and inocula at phylum level. Age when sacrificed in days is shown for each control or contact chick. SE–S. Enteritidis infected chickens. *S*. Enteritidis infection is of minimal effect on microbiota composition in chickens not detectable at phylum level [13, 14]. Aviguard, WCHA and YCFA inocula show microbiota composition of the Aviguard or washes from appropriate agars.

Next we tested whether the same microbiota development can be achieved by administration of *in vitro* subcultured gut anaerobes. The microbiota of chicks inoculated with Aviguard or bacterial washes from WCHA and YCFA agars differed from the control, non-inoculated chicks (Fig 3). Their microbiota was enriched for *Bacteroidetes* and *Actinobacteria*, similar to the chicks raised in contact with a hen. *Bacteroidetes* in these chicks formed around 60% of the total microbiota, *i*.*e*. even more than in the chicks raised with contact hens (compare Figs 2 and 3).

### Complex microbiota reduce chick colonization with S. Enteritidis

**Fig 4:**
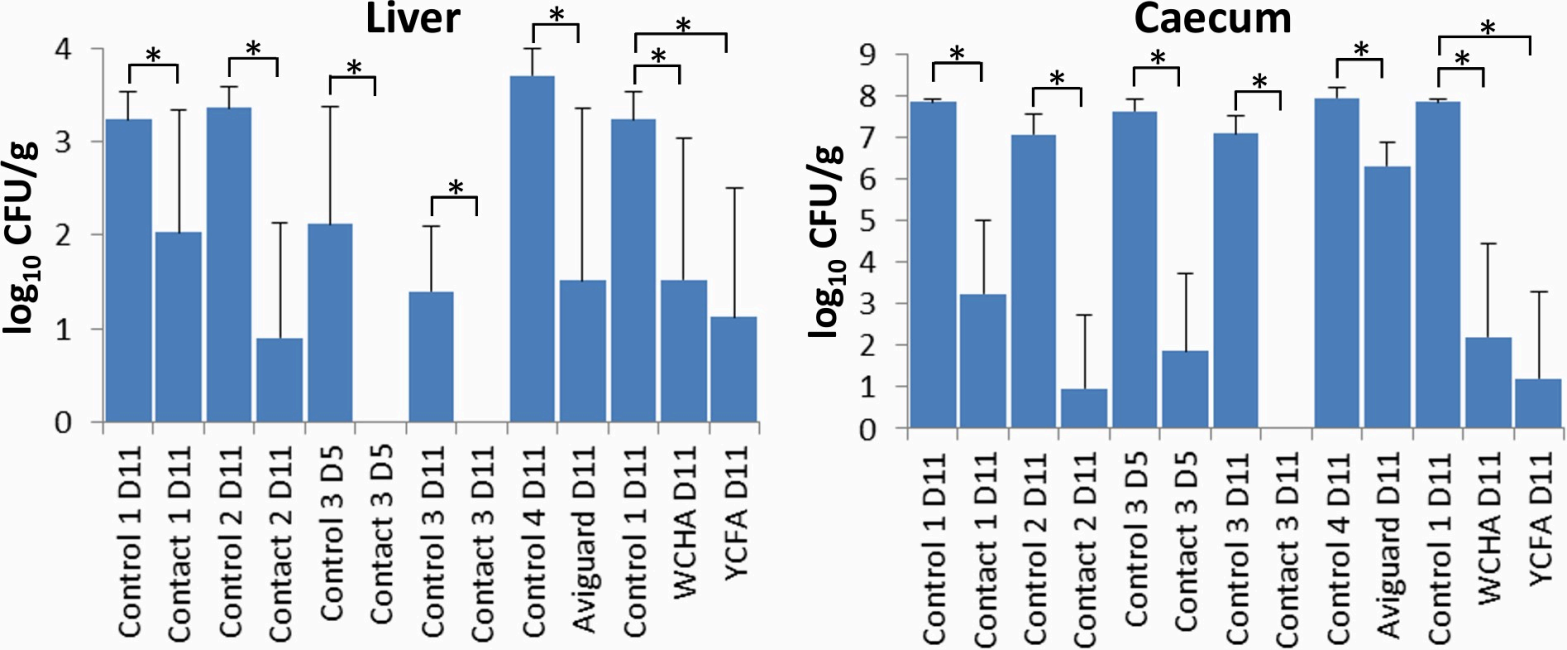
Enteritidis counts in the liver and caecum of control, contact or microbiota inoculated chicks. ***S*.** Control and contact chicks labelled as 1–3 belong to the three experiments with contact hens. “Control 4” chicks were used in the experiment with Aviguard. Since chicks inoculated with washes from WCHA and YCFA agars were parts of experiment 1 with the contact hen, their appropriate control chickens are therefore identified as Control 1. *—p<0.05 by t-test.

Accelerated development of chicken gut microbiota in all experiments significantly increased the chicken’s resistance to *S*. Enteritidis infection. Resistance to caecum colonisation by *S*. Enteritidis increased more than 5 logs in contact chicks compared to controls, around 2 logs in Aviguard treated chicks, and around 6 logs in the chicks inoculated with bacterial washes from WCHA or YCFA agars (Fig 4). The onset of protection occurred within 24 hours after inoculation as shown in experiment 2 with the contact hen, in which the chicks sacrificed on day 5 were challenged with *S*. Enteritidis on day 2 of life. *S*. Enteritidis counts in the liver confirmed the data from the caecum.

### Identification of bacterial genera transferred from hens to contact chicks

Thirteen genera were passed defined criteria and these included genera *Bifidobacterium* and *Olsenella* (both belonging to *Actinobacteria*), *Bacteroides*, *Barnesiella*, *Parabacteroides*, *Paraprevotella*, *Prevotella* and *Alistipes* (all from phylum *Bacteroidetes*), *Desulfovibrio* (*Proteobacteria*), *Mucispirillum* (*Deferribacteres*), *Faecalibacterium* (*Clostridiales*/*Firmicutes*) and *Phascolarctobacterium* and *Megamonas* (both *Selenomonadales*/*Firmicutes*). When summed up, these genera formed 44.78% of all microbiota in contact chicks but only 1.44% of all microbiota in control chicks (Table 1). Hens therefore acted as an important source of these genera for newly hatched chicks.

**Table 1:** Bacterial genera which were more abundant in microbiota of chicks raised in a contact with an adult hen than in the control chicks.

Phylum	Family	Genus	Control (%)*	Contact (%)*	Contact/ Control^#^
Actinobacteria	Bifidobacteriaceae	Bifidobacterium	0.067	1.714	25.6
Actinobacteria	Coriobacteriaceae	Olsenella	0.038	0.651	17.1
Bacteroidetes	Bacteroidaceae	Bacteroides	0.454	9.383	20.7
Bacteroidetes	Porphyromonadaceae	Barnesiella	0.085	10.023	118.3
Bacteroidetes	Porphyromonadaceae	Parabacteroides	0.071	1.731	24.4
Bacteroidetes	Prevotellaceae	Paraprevotella	0.068	2.140	31.5
Bacteroidetes	Prevotellaceae	Prevotella	0.047	1.993	42.8
Bacteroidetes	Rikenellaceae	Alistipes	0.032	2.756	86.3
Deferribacteres	Deferribacteraceae	Mucispirillum	0.014	0.505	36.9
Firmicutes	Ruminococcaceae	Faecalibacterium	0.191	6.687	35.0
Firmicutes	Acidaminococcaceae	Phascolarctobacterium	0.100	2.224	22.2
Firmicutes	Veillonellaceae	Megamonas	0.190	4.301	22.6
Proteobacteria	Desulfovibrionaceae	Desulfovibrio	0.079	0.675	8.5
Sum			1.44	44.78	31.2

* average abundance of given genus in microbiota of control or contact chicks

# ratio of abundance in contact and control chick microbiota

### Bacterial genera of decreased abundance in microbiota of contact chickens

Five genera passed defined criteria (Table 2) and these included genera *Blautia*, *Anaerostipes* and *Clostridium* XIVa (all family *Lachnospiraceae*/phylum *Firmicutes*) and *Proteus* and *Escherichia* (both phylum *Proteobacteria*). We noticed that the abundance of genera belonging to family *Lachnospiraceae* decreased approx. 10 fold whilst *Proteobacteria* decreased approx. 40 fold in microbiota of contact chickens. Microbiota transferred from adult hens to offspring (Table 1) was therefore of higher suppressive effect on *Proteobacteria* than on the representatives of *Lachnospiraceae*/*Firmicutes*.

**Table 2:** Bacterial genera which were more abundant in microbiota of control chicks than in the chicks raised in contact with an adult hen.

Phylum	Family	Genus	Control (%)*	Contact (%)*	Control/ Contact^#^
Firmicutes	Lachnospiraceae	Blautia	6.780	0.476	14.2
Firmicutes	Lachnospiraceae	Anaerostipes	0.803	0.117	6.9
Firmicutes	Lachnospiraceae	Clostridium XIVa	17.625	3.053	5.8
Proteobacteria	Enterobacteriaceae	Proteus	1.942	0.042	45.8
Proteobacteria	Enterobacteriaceae	Escherichia	10.168	0.264	38.6
Sum			37.32	3.95	9.4

* average abundance of given genus in microbiota of control or contact chicks

# ratio of abundance in control and contact chick microbiota

### Inoculation of newly hatched chicks with pure cultures of selected anaerobes

**Fig 5:**
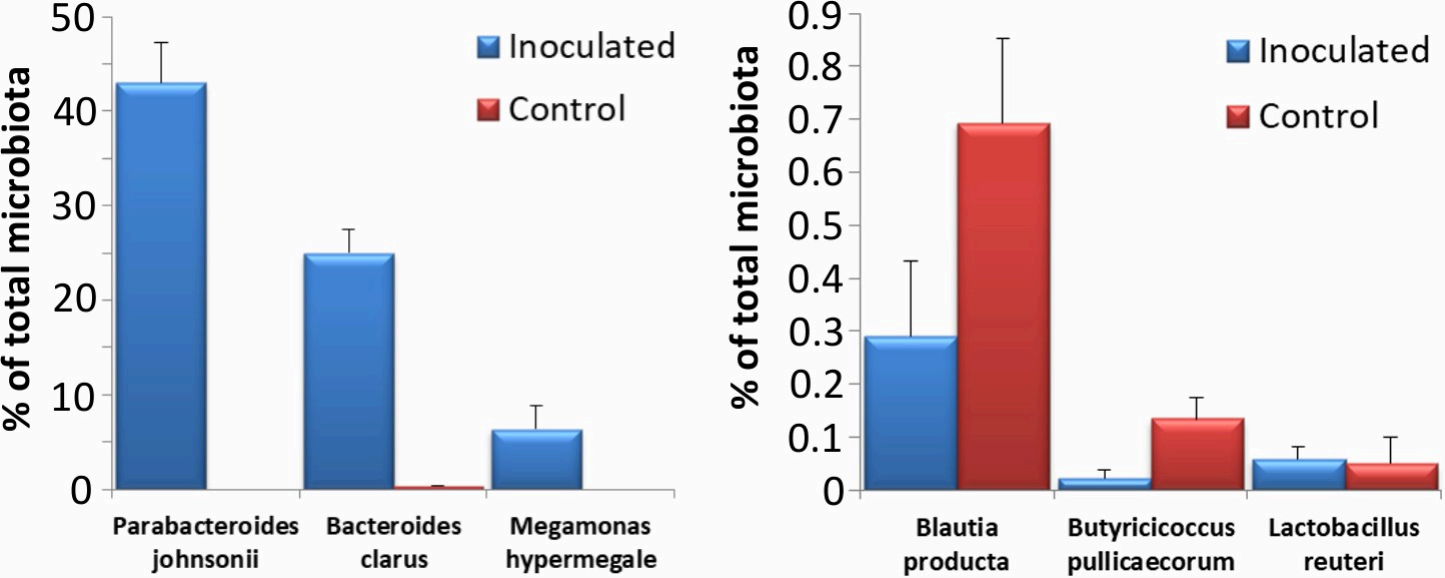
Ability of 6 selected gut anaerobes to colonise the chicken caecum. Chicks were orally inoculated with bacterial species as indicated and 7 days later their presence in the caecum in the inoculated and control chickens was determined by sequencing of 16S rRNA genes. *Parabacteroides johnsonii*, *Bacteroides clarus* and *Megamonas hypermegale* efficiently colonised the chicken caecum while the abundance of *Blautia producta*, *Butyricicoccus pullicaecorum* and *Lactobacillus reuteri* in inoculated and control chicks did not differ. Inoculation with the latter three isolates, unlike the former three, did not result in efficient caecum colonisation.

To confirm previous findings on ability or inability of particular taxons to colonise the caecum of chicks during the first week of life, the newly hatched chicks were finally inoculated with pure cultures of *Parabacteroides johnsonii*, *Bacteroides clarus*, *Megamonas hypermegale*, *Butyricicoccus pullicaecorum*, *Blautia producta* and *Lactobacillus reuteri*. Based on previous results we expected that *P*. *johnsonii*, *B*. *clarus* and *M*. *hypermegale* would colonise while *B*. *pullicaecorum*, *Bl*. *producta* and *L*. *reuteri* would not. When caecal contents collected on day 8 of life were subjected to microbiota characterisation by 16S rRNA gene sequencing, *P*. *johnsonii*, *B*. *clarus* and *M*. *hypermegale* efficiently colonised the chicken caecum and formed 43.0%, 25.0%, or 6.4% of caecal microbiota, respectively (Fig 5). On the other hand, inoculation of chicks with *B*. *pullicaecorum*, *Bl*. *producta*, or *L*. *reuteri* did not result in caecal colonisation and their abundance both in the inoculated chicks and control chicks was lower than 1% of total microbiota (Fig 5).

## Discussion

**Fig 6:**
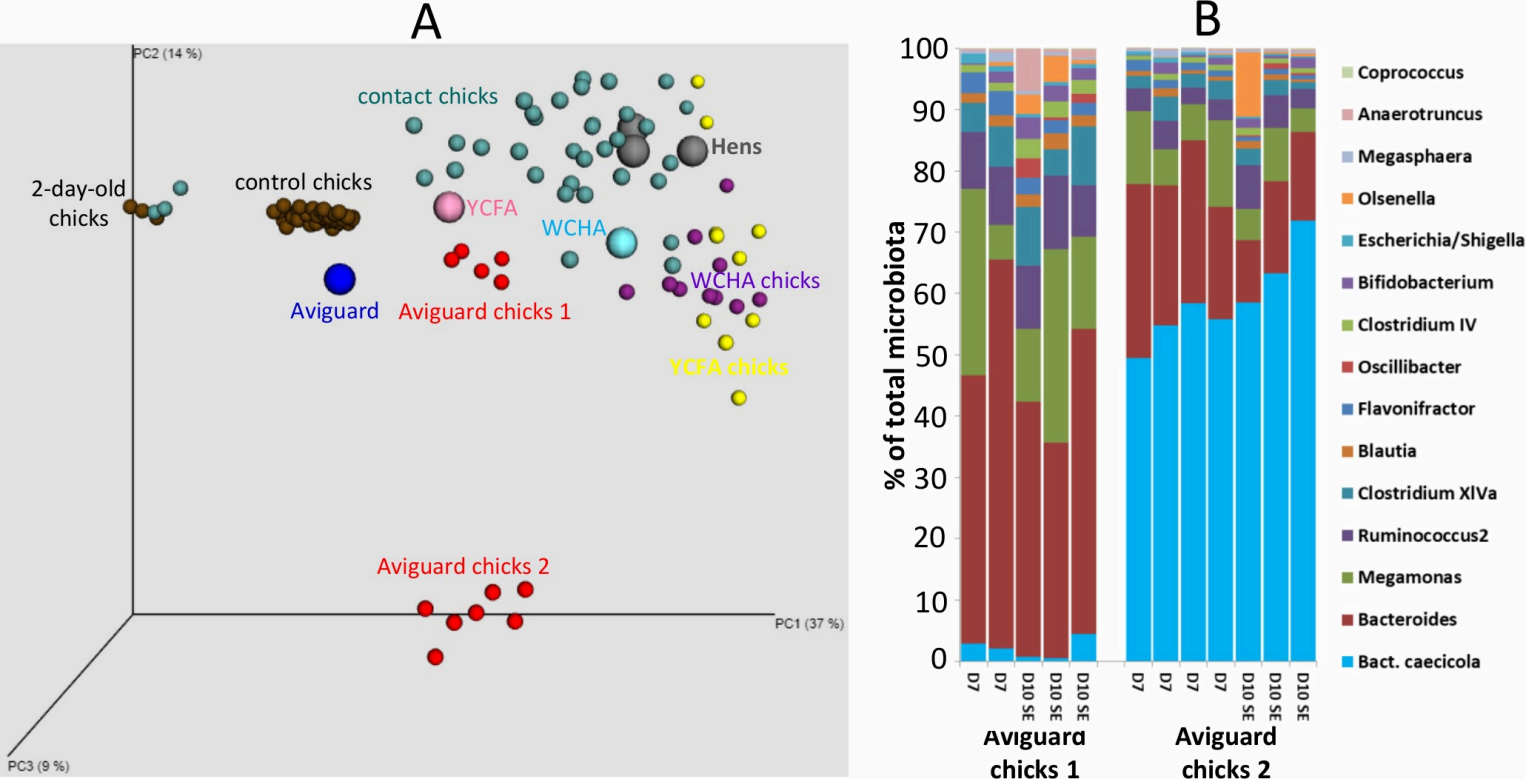
Caecal microbiota composition in newly hatched chicks is affected by available sources. Panel A, PCoA analysis of all samples processed in this study. Hen, Aviguard, WCHA and YCFA wash samples as microbiota sources are highlighted with larger spots. Two-day-old control and contact chicks formed a separate cluster which means that their microbiota composition differed from the rest of the chicks and hens. The remaining control chicks from all experiments aged 5 to 11 days formed another cluster. Hens, contact chicks, or Aviguard, WCHA and YCFA washes inoculated chicks formed the last cluster except for 7 chickens treated with Aviguard. These 7 chicks were highly colonised by *Bacteroides caecicola* as shown in the panel B, and this colonisation was independent of age (shown in days D) or infection with *S*. Enteritidis (*S*. Enteritidis chicks are identified as SE).

In this study we addressed the basic principles of caecal microbiota development in chicks during the first two weeks of life since the correct colonisation of the intestinal tract considerably increases chicken resistance to pathogen colonisation [4, 6]. We have repeatedly shown that the differences between commercially hatched and raised chicks and chicks in contact with adult hens were quite extensive. Microbiota of chicks raised in the presence of an adult hen developed quickly and within a week reached a composition similar to that observed in adult birds (Fig 6A). A mere 24-hour-long contact between the chicks and a hen was long enough for their inoculation and seeding although a few additional days were needed (more than 1 but less than 3 days) before the microbiota completely developed. Moreover, when the chicks were administered mixtures of moderate complexity, microbiota members similar to those transferred by contact efficiently colonised chicken caecum. These observations have several consequences. First, microbiota development, which we described earlier [2], will be considerably affected by microbiota sources. Secondly, studies on gut microbiota performed in young chicks in extremely hygienic experimental settings will more frequently encounter Gram-positive *Firmicutes* than studies performed in chicks from commercial settings with less controlled conditions. Thirdly, we cannot exclude that for some bacterial species the 7 or 11-day-long window for which we monitored microbiota development was not long enough to allow them to reach detectable abundance. An important time point may occur before and after week 2 of life when B-lymphocytes infiltrate the gut mucosa and chickens start to express their own mucosal antibodies [15–17] and some microbiota members may appear or disappear after this time point. Fourth, experiments with randomly selected donor hens are always dependent on their microbiota composition. This means that there might be additional bacterial genera which can colonise chicks during their first days of life but if these were underrepresented or absent in one or all donor hens used in this study, we could have missed them. We have unpublished data showing that *Campylobacter* and *Helicobacter* (both *Epsilonproteobacteria*), *Megasphaera* and *Veillonella* (both *Veillonellaceae*), or *Akkermansia* (*Verrucomicrobia*) and *Fusobacterium* (*Fusobacteria*) can be also transferred from hens to chicks.

All experiments showed Gram-negative bacteria were usually easily transferrable. We observed successful transfer of numerous genera from phylum *Bacteroidetes* but also representatives of phyla *Deferribacteres* or *Proteobacteria*. Similar results were recorded also by Impey et al. who used mixed cultures for oral inoculation of chicks more than 35 years ago and proposed *Bateroides* sp. as a suitable marker of successful colonisation [18]. Despite this, not every Gram-negative species can be transferred from hens to offspring as could be seen in the Aviguard treated chicks. These chicks split into two groups due to the varying abundance of *Bacteroides caecicola*. This bacterium did not extensively colonise group 1 of Aviguard-treated chicks (average representation was 1.9%) while *B*. *caecicola* formed 55.8% of total microbiota in group 2 Aviguard-treated chicks (Fig 6B). Since all Aviguard-treated chicks were kept in the same space, the chicken separation effect can be excluded and there must have been other factor(s), *e*.*g*. chicken genetics, which determined the chicken’s competence for colonisation with *B*. *caecicola*.

The ability of Gram-positive bacteria to effectively colonise newly hatched chicks was more complex. Representatives of *Actinobacteria* were transferred from hens to chicks in all 3 contact experiments and could be transferred to newly hatched chicks also by Aviguard or agar plate washes. Despite this, representatives of *Actinobacteria* never reached high abundance as observed for the genera belonging to the phylum *Bacteroidetes*. Chicks could be colonised also with representatives of order *Selenomonadales*, genera *Megamonas* or *Phascolarctobacterium*. *Selenomonadales*, despite being phylogenetically related to Gram-positive *Firmicutes*, harbour genes for the expression of Gram-negative cell wall type [19]. Whether this is relevant for their ability to colonise the intestinal tract of newly hatched chicks will have to be determined though the association of outer membrane and resistance to bile salts is well known, and bile salts are present in many selective agars for suppression of Gram-positive bacteria. Rather surprisingly, except for *Faecalibacterium*, the rest of the representatives of phylum *Firmicutes* was impossible to transfer. This is valid for *Lactobacilli*, but also for common gut microbiota members belonging to families *Lachnospiraceae* or *Ruminococcaceae*. The reasons for the inability to colonise are currently being intensively studied in our lab. One of the possible explanations for *Lachnospiraceae* or *Ruminococcaceae* (but not *Lactobacilli*) is that their life cycle might be dependent on spore formation whilst preparations which we used for chick inoculation were enriched for vegetative cells. The importance of spores for the life cycle of *Firmicutes* may indirectly explain why *Faecalibacterium* was transferred from hens to chicks since *Faecalibacterium* does not form spores. Though in an apparent contradiction, these specific characteristics could have led to selection of alternative mechanisms by which *Faecalibacterium* spread in animal populations. In fact, this has already been noticed in humans [20].

Since the microbiota provided to contact chicks by a hen formed nearly 50% of total caecal population (Table 1), the abundance of *Firmicutes* and *Proteobacteria* (Table 2) should apparently decrease to half due to percentage calculations, if there are no additional interactions. However, microbiota members belonging to family *Lachnospiraceae* decreased approx. 9 fold, and *E*. *coli* and *Proteus* decreased approx. 40 fold (Table 2). This means that microbiota transferred from hens to chicks is of extra negative selection against strains belonging to family *Enterobacteriaceae* but less suppressive towards strains belonging to *Lachnospiraceae*.

In this study we addressed the issue of microbiota transfer and development in newly hatched chicks. We have shown that caecal microbiota development is different in chicks and chicks raised with or without contact with an adult hen. Microbiota transfer is quick since 24-hour long contact between donor hen and chicks was long enough for their seeding. *Bacteroidetes*, *Actinobacteria*, *Selenomonadales* and *Faecalibacterium* were efficiently transferred from donor hens to chicks. However, we never recorded the transfer of *Lactobacilli* or *Clostridiales*. These conclusions should be considered when designing the next generation of probiotics or when performing faecal microbiota transplantations as tested earlier [18, 21]. Although *Lactobacilli* or *Clostridiales* may affect the development of the intestinal tract by merely passing through it, the positive effect of probiotics on gut health will likely increase with ability of probiotic bacteria to succesfully colonise.

## Supporting information

S1 Table OTU table of all samples processed in this study. (XLS)
